# Differences on photosynthetic limitations between leaf margins and leaf centers under potassium deficiency for *Brassica napus* L.

**DOI:** 10.1038/srep21725

**Published:** 2016-02-23

**Authors:** Zhifeng Lu, Tao Ren, Yonghui Pan, Xiaokun Li, Rihuan Cong, Jianwei Lu

**Affiliations:** 1 College of Resources and Environment, Huazhong Agricultural University, Wuhan 430070, China; 2 Key Laboratory of Arable Land Conservation (Middle and Lower Reaches of Yangtze River) Ministry of Agriculture, Wuhan 430070, China

## Abstract

Analyzing the proportions of stomatal (S_L_), mesophyll conductance (MC_L_) and biochemical limitations (B_L_) imposed by potassium (K) deficit, and evaluating their relationships to leaf K status will be helpful to understand the mechanism underlying the inhibition of K deficiency on photosynthesis (*A*). A quantitative limitation analysis of K deficiency on photosynthesis was performed on leaf margins and centers under K deficiency and sufficient K supply treatments of *Brassica napus* L. Potassium deficiency decreased *A*, stomatal (*g*_s_) and mesophyll conductance (*g*_m_) of margins, S_L_, MC_L_ and B_L_ accounted for 23.9%, 33.0% and 43.1% of the total limitations. While for leaf centers, relatively low limitations occurred. Nonlinear curve fitting analysis indicated that each limiting factor generated at same leaf K status (1.07%). Although MC_L_ was the main component of limitations when *A* began to fall, B_L_ replaced it at a leaf K concentration below 0.78%. Up-regulated MC_L_ was related to lower surface area of chloroplasts exposed to intercellular airspaces (*S*_c_/*S*) and larger cytosol diffusion resistance but not the cell wall thickness. Our results highlighted that photosynthetic limitations appear simultaneously under K deficiency and vary with increasing K deficiency intensity.

Potassium (K), one of the macronutrients essential for plant growth and development, is involved in many physiological processes, such as photosynthesis, enzyme activation, water relations, assimilate transport, and protein synthesis^1^,^2^. K deficiency profoundly decreased crop yield^3^,^4^, thus strategies of survival and improvement would be important for plant growing under adverse conditions. It is a truism that most of the dry matter is formed by leaf photosynthesis (*A*) which is intimately connected with K status. Multifarious studies have come to a nearly consistent conclusion that leaf *A* decreases in K-starved plants^3^,^5^,^6^, therefore an even deep comprehending of mechanism underlying the inhibition of K deficiency on *A* is necessary^2^.

During photosynthesis, CO_2_ moves from external atmosphere to the internal leaf air spaces through the stomata, and from there to carboxylation sites inside the chloroplasts^7^. It is established that stomatal closure is the foremost limitation to CO_2_ assimilation due to the vital role of K in stomatal aperture^8^,^9^. As a major osmotica, K^+^ accumulation in vacuole is essential for stomatal opening, which had been verified to be initially dropped under K deficiency^10^. And because of this, Bednarz *et al.*^5^ stated that the most limiting resistance to *A* of *Gossypium hirsutum* L. came from stomata^5^. In contrast, K starvation caused a decreased *A* and stomatal conductance (*g*_s_), but an increased intercellular CO_2_ concentration (*C*_i_) of *Carya cathayensis* leaves, suggesting that, in addition to *g*_s_, mesophyll conductance (*g*_m_) and biochemical limitations might be involved in the depression of photosynthesis in K deficient conditions^6^. Numerous studies have shown that *g*_m_ is relatively low, leading to great draw-down of chloroplastic CO_2_ concentration from *C*_i_, and changed along the variation of water status, nitrogen nutrient, irradiance, temperature and CO_2_ concentration^11^,^12^,^13^,^14^. Moreover, leaf structures, specific aquaporins, plasma membrane etc. are involved in the determinations of *g*_m_^15^. K starvation might have reduced aquaporin activity^16^ and increased leaf dry mass per unit area (*M*_A_)^1^,^17^, therefore, causing a stronger mesophyll diffusion resistance to CO_2_ delivery^18^. Besides, K nutrition also known to increase the leaf intercellular air space to enhance *g*_m_^1^.

Additionally, biochemical processes may restrain photosynthesis, particularly under severe and/or long-time K starvation^1^,^5^,^6^. It was reported that Rubisco (ribulose-1,5-bisphosphate carboxylase/oxygenase, EC 4.1.1.39) activity was decreased under K deficiency, becoming a major limiting factor for photosynthesis in *Oryza sativa* leaves^3^. Chlorophyll synthesis was observed to be significantly impaired under K deficiency in *Eucalyptus grandis* leaves^1^. Moreover, K starvation up-regulated the fraction of electron transport to O_2_, resulting in an increased reactive oxygen species (ROS)^19^. Carbohydrate accumulation which may feedback regulation of leaf photosynthesis is more easily observed in K starved leaves^20^,^21^. Indeed, the relative contributions of these three limiting processes to photosynthesis under K deficiency and the underlying mechanisms have not been fully explored, due to the complicated physiological processes and variation of dominant limiting factors under differ K deficiencies^2^,^5^. No matter what the primary cause of decrease *A*, the discrepancy between researches was believed to be derived from differ physiological K deficiency severities. For this reason, a comprehensive consideration of whole limiting factors and their relationships with leaf K status seems to be important.

In 2005, Grassi and Magnani proposed a method to accurately quantify photosynthetic limitations by separating the relative controls on *A* resulting from S_L_, mesophyll conductance (MC_L_) and biochemical limitations (B_L_)^22^. This method has been successfully applied for evaluating the relative control of leaf *A* under water stress and during their recovery processes, among inter- and intra-species^13^,^23^,^24^,^25^. It showed not only great potential for elucidating the magnitude changes of limitations and their dominance in photosynthetic restraints with increasing severity of K deficiency, but also revealing the corresponding critical K concentrations for their transformation.

Winter oilseed rape (*Brassica napus* L.), a model-plant of winter cover crops who needs substantial amount of potassium to growth was used for a deeply aggregate analysis of K deficiency on photosynthetic limitations^26^. However, malfunction of physiological processes like photosynthesis is hard to be affected when K concentration above the threshold value (1.5% in dry matter, or less)^10^. On consideration of the fact that potassium deficiency symptoms, characterized by a chlorosis and even scorch around the periphery can be obviously observed when leaf K concentration below 1.0% in most species^27^. And the withdrawal K initially occurred at the edge of leaf tip, as tip cells are initially proliferated and oldest^28^, resulting in different K levels as well as visible distinctions between centers and margins. These natural K gradients are therefore precious for photosynthetic limitation analysis, from which we may seek out the main limiting factors under variable leaf K status and the corresponding threshold values. This phenomenon occurred more frequently under a complex biological and abiological environment system during a long-time and low-temperature wintertide, which may conducive to generate a physiology K deficiency in a K-deficient soil, i.e., it may bring K function into full play^29^,^30^. Accordingly, the objectives of the present study were to: (1) estimate the differences of contributions for three limiting factors to photosynthesis between leaf margins and leaf centers, (2) uncover the relationships between photosynthetic limitations and diminishing leaf K status, and therefore the critical K concentration for the predominate restraint transformation, (3) reveal the mechanism underlying the K-induced variation of limiting factors. It is hoped that this research will facilitate a better understanding of the photosynthetic physiological mechanism by which potassium deficiency leads to growth retardation in oilseed rape.

## Results

### Plant performance, leaf K concentration and net photosynthesis

The total dry matter of the –K treatment decreased significantly by 29.9% on average versus the +K treatment (Table 1). The leaf expansion was also restrained, with a 22.1% and 18.0% decline in the individual leaf dry matter and leaf area, respectively. Leaf K concentration was dramatically influenced by potassium supply and leaf position, which was significantly lower in the –K treatment than in the +K treatment. Meanwhile, within an individual leaf, K concentration was remarkably lower in margins than in centers. The mean net photosynthesis (*A*) in the leaf margins of the –K treatment was 56.9% that of the +K treatment. However, there was no significant difference between leaf margins and centers under the –K treatment, as well as the two positions under the +K treatment.

### Stomatal conductance

Potassium deficiency led to a significant decline of the mean stomatal conductance (*g*_s_) in leaf margins, which was 63.6% that of the +K treatment. However, the mean *g*_s_ value of the leaf centers was not influenced by K nutrient (Table 2). There was a significantly lower *g*_s_ in leaf margins than in leaf centers under the –K treatment, whilst the *g*_s_ values of these two positions were the same under the +K treatment. Despite a decrease of the *g*_s_ value in leaf margins of the –K treatment, the intercellular CO_2_ concentrations (*C*_i_) value was raised, and the mean *C*_i_ were similar to those of other groups.

Potassium supply and leaf position had no effects on stomatal frequency and stomatal length (Table 2). However, stomatal width was significantly decreased in the –K treatment, especially in the leaf margins where the width decreased by 20.9% as compared with the +K treatment. Stomatal pore area was therefore considerably decreased due to K deficiency, particularly in the leaf margins with a 28.0% decline in the single stomatal pore area. Nevertheless, the stomatal length and width as well as the stomatal pore area showed no significant difference in these two positions under the +K treatment.

### Mesophyll conductance

Despite a dramatic decrease in the mean mesophyll conductance (*g*_m_) in the leaf margins of the –K treatment, the mean chloroplastic CO_2_ concentrations (*C*_c_) was 9.4% higher than that of the +K treatment (Table 3). The mean *g*_m_ and *C*_c_ values were similar in the leaf centers of different K treatments, as well as between the two positions in the +K treatment. Potassium niutrient and leaf position did not affect mean intercellular CO_2_ compensation point (*C*_i_^*^) and mitochondrial respiration rate in the light (*R*_d_), however, the mean chloroplastic CO_2_ compensation point (Γ*) was significantly increased in leaf margins of the –K treatment, but showed no statistical differences among the other three groups.

### Biochemical characteristics

The mean maximum rate of electron transport (*J*_max_) and maximum rate of carboxylation (*V*_c,max_) in the leaf margins of the –K treatment were the lowest, and minor changes were observed among the other three treatments (Table 4). However, the mean *J*_*max*_/*V*_*c,max*_ in leaf margins of the –K treatment was dramatically increased compared with the mean values of the other three groups in the range from 1.42 to 1.46. The variation of photosynthetic parameters was verified by chemical analyses (Table 4). A significant decline of leaf chlorophyll concentration was found in the –K treated leaves, especially in the leaf margins, with a 31.1% decrease. Furthermore, Rubisco activity was dramatically decreased in leaf margins of the –K treatment, but it was the same in the leaf centers of the –K treatment and the two positions of the +K treatment. Potassium deficiency caused severe ROS production in leaf margins where O_2_^.−^ generation rate increased by 22.8%, and meanwhile, POD activity increased by 25.5%.

### The relationship between relative *A*, *g*_s_ and *g*_m_ with leaf K concentration

**Figure 1:**
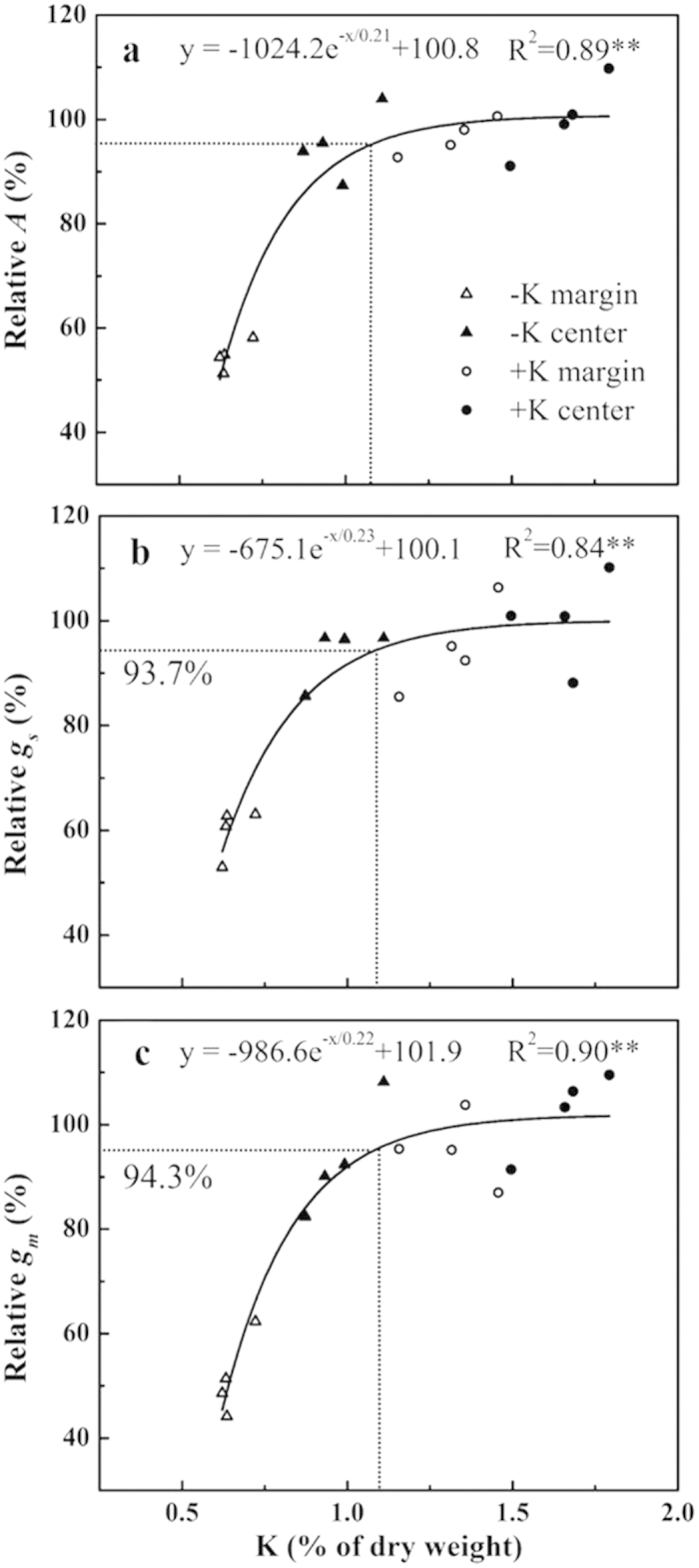
Relationship between relative photosynthetic parameters and leaf K concentration. Relative (**a**) photosynthesis rate (*A*), (**b**) stomatal conductance (*g*_s_), (**c**) mesophyll conductance (*g*_m_). The values were the relative proportion of measured values over the mean values of K-sufficient leaf centers. Each point represents one leaf measurement. Open triangles and closed triangles represent the values of leaf margin and leaf center under the –K treatment, while open circles and closed circles represent those of the +K treatment. Equations, regression coefficients, and significance are shown when *P* ≤ 0.05 (*P ≤ 0.05; **P ≤ 0.01).

A significant curvilinear relationship between relative *A*, *g*_s_ or *g*_m_ and leaf K concentrations is shown in Fig. 1. The relative values increased with increasing leaf K concentration, and remained stable when the leaf K concentration was beyond a certain concentration. Here a photosynthesis-based concentration threshold with the relative values reaching 95.0% of the maxima was defined. The relative *A* values increased rapidly with increasing leaf K concentration when it was less than 1.07% (Fig. 1a), and varied little when the leaf K concentration was above 1.07%. Therefore, the K concentration (1.07%) was used to evaluate the relative *g*_s_ and *g*_m_ (Fig. 1b,c), and the calculated result (93.7% and 94.3%) was close to 95.0%, indicating that this threshold value was acceptable for *g*_s_ and *g*_m_.

### Quantitative limitation analysis

**Figure 2:**
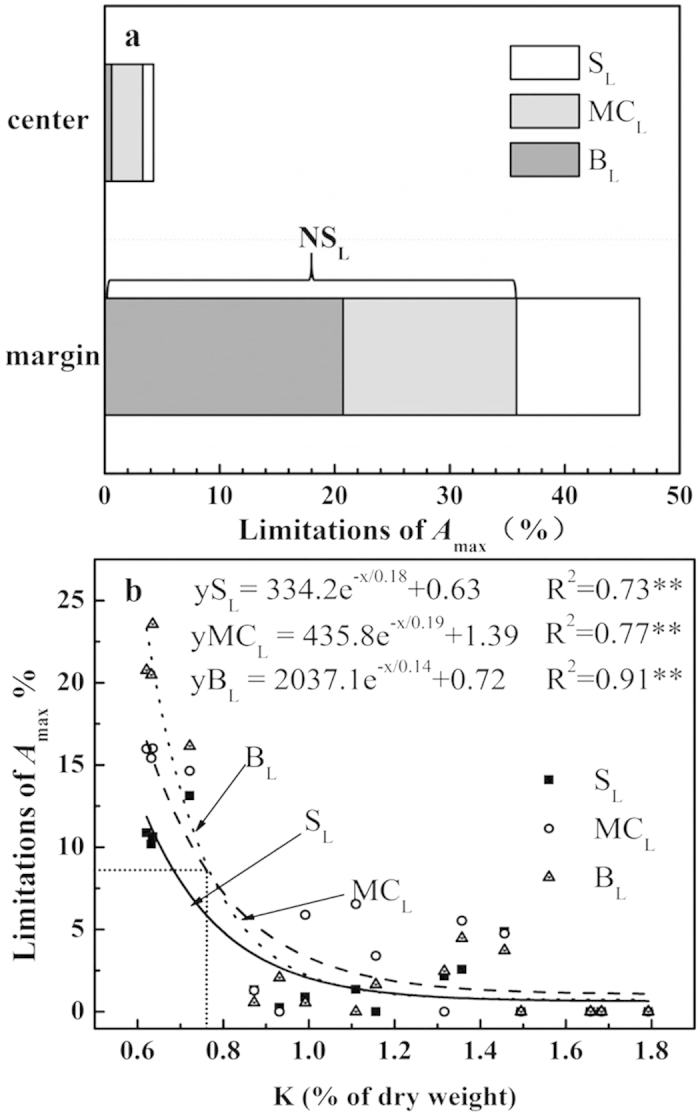
Photosynthetic limitations and their response to leaf K concentration. (**a**) Quantitative limitation analysis of photosynthetic CO_2_ assimilation in leaf margins and centers under the –K treatment. Values are mean ± SE of four replicates per position. The open, gray, and dark gray bars represent the percentages of stomatal (S_L_), mesophyll conductance (MC_L_), and biochemical (B_L_) limitations, respectively. (**b**) Relationships between limitations and leaf K concentration. Each point with the same shape represents a single leaf (n = 16). The symbols are as follows: S_L_, closed squares; MC_L_, open circles; B_L_, closed triangles. Solid, dash, and dot lines are regression curves of S_L_, MC_L_, and B_L_, respectively. Equations, regression coefficients, and significance are shown when *P* ≤ 0.05 (*P ≤ 0.05; **P ≤ 0.01).

The restrictions of *A*_max_ in the –K leaves posed by stomatal (S_L_), mesophyll conductance (MC_L_) and biochemical limitations (B_L_) are presented in Fig. 2a. In symptomatic margins, total limitations reached a value of 46.9%, and the contribution of S_L_, MC_L_ and B_L_ represented 23.9%, 33.0% and 43.1% of total limitations, respectively. By contrast, despite the relatively low limitation (4.8%) in the leaf center, MC_L_ contributed a primary limitation to *A*_max_. Accordingly, the dominant limitations changed from symptomatic leaf margins to centers. The relationship between relative limitations and leaf K concentrations were further analyzed (Fig. 2b). All the limitations declined precipitously with the leaf K concentration increased from 0.6 to 1.07% (according to the K-based concentration threshold), particularly the B_L_ with the maximum slope of the fitted curve, but they gradually decrease as leaf K concentration continues to increase. Their relative contribution also varied with the change of the leaf K status. While MC_L_ largely predominated at the leaf K concentration of less than 1.07%, B_L_ replaced it when the K concentration was below 0.78% (leaf K concentration of the intersection point between B_L_ and MC_L_ fitted curves).

## Discussion

### Limitations imposed by K deficiency occur at the same time

In the present study, *A* in leaf margins were weakened by K deficiency. Generally, the declining *A* is considered to be limited by stomatal and mesophyll resistances to CO_2_ diffusion, and biochemical obstacles^13^,^22^. Here we demonstrated that *g*_s_, *g*_m_ and biochemical activities were profoundly restricted as *A* down-regulated. Stomatal conductance which determine the vital step of CO_2_ diffuse from the atmosphere to the interior of leaf was markedly decreased in –K leaf margins, as reported for *Eucalyptus grandis*^1^, *Gossypium hirsutum*^17^, and *Oryza sativa*^3^. This is mainly because the lack of vacuole K to keep stomatal aperture by providing driving force to promote water inpour into the guard cell vacuole^31^. The declined *A*, to a certain extent, revealed that the K in cytoplasm identified as biochemical functional component was below the critical value^10^. Therefore, malfunction of physiological process could come with limited *A*.

**Figure 3:**
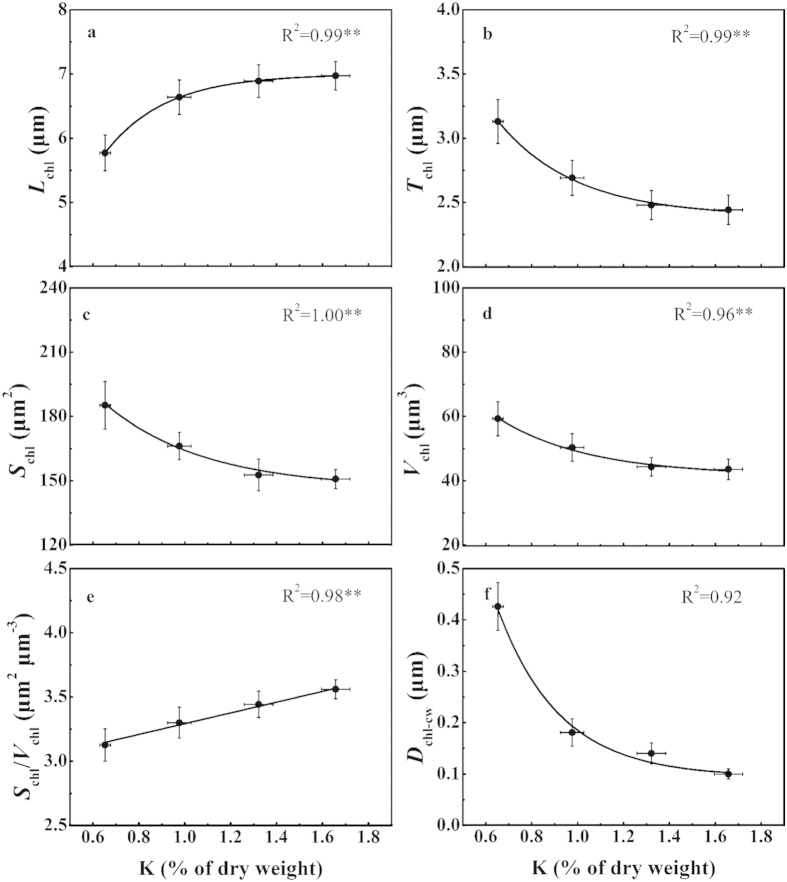
The relationship between chloroplast characteristics and leaf K concentration. (**a**) Chloroplast length (*L*_chl_), (**b**) thickness (*T*_chl_), (**c**) surface area (*S*_chl_), (**d**) volume (*V*_chl_), (**e**) *S*_chl_/*V*_chl_, (**f**) distance of chloroplast from the cell wall (*D*_chl-cw_). Values are mean ± SE of four replicates for K concentration and at least thirty replicates for microstructure parameters. Regression coefficients and significance are shown when *P* ≤ 0.05 (*P ≤ 0.05; **P ≤ 0.01).

Likewise, *g*_m_ was decreased in parallel with *A*. Indeed, *g*_m_ might be down-regulated by increasing leaf dry mass per area (*M*_A_)^7^,^23^, however, in the present study, there was no remarkable difference in *M*_A_ between the –K and +K leaves (Table 5; Supplementary Fig. S1). Cell wall thickness (*T*_cell-wall_) and surface area of chloroplasts exposed to intercellular airspaces (*S*_c_/*S*) are reported to be the most substantial anatomical traits in determining *g*_m_^23^,^32^. However, significant differences in mesophyll cell wall surface area exposed to intercellular airspace per leaf area (*S*_m_/*S*) and *S*_c_/*S*, but not *T*_cell-wall_ between leaf margins of two K treatments were observed (Table 5). Besides, chloroplast size^33^ has also been proved to influence *g*_m_. In the present study, though the chloroplast length (*L*_chl_) decreased under lowest K status, the thickness (*T*_chl_), surface area (*S*_chl_) and volume (*V*_chl_) of chloroplast were largely increased, however, the *S*_chl_/*V*_chl_ was smaller (Fig. 3). The chloroplast enlarging under lowest K concentration was not completely same to that discovered under low nitrogen conditions^33^,^34^. The increase of *T*_chl_ was more likely to be based on the sacrifice of length owing to roughly circular envelope (Fig. 3a,b; Supplementary Fig. S2). Mathematically, ellipsoidal chloroplasts, combining with an increscent chloroplast number (see Supplementary Fig. S3) were more probably to have longer length of chloroplasts facing the cell wall than swollen even sphere envelopes. Furthermore, the resistance along diffusion pathway length in cytoplasm (distance of chloroplast from cell wall, *D*_chl-cw_) and stroma (taken as half of the chloroplast thickness) account for 10–50% of *g*_m_ limitation^23^, which however, reported only up to 22% of liquid phase resistance (*r*_liq_) by Evans *et al.* in 1994^35^. Low K status strongly increased *T*_chl_ and *D*_chl-cw_ (Fig. 3b,f), accordingly, the corresponding resistance would be increased. It is therefore proved that the decreased *g*_m_ is primary due to the reduced *S*_c_/*S* and larger cytosol diffusion resistance but not *T*_cell-wall_. More evidences may seek from the influence of K on plasma membrane and chloroplast envelope conductance^32^, carbonic anhydrase and aquaporins that participated in determination of *g*_m_^14^,^15^,^23^.

It should be noted that *A*, *g*_s_ or *g*_m_ started to decline almost at the same time with an extremely similar leaf K status. By another way, the quantitative analysis of limitations indicated that three limiting factors coexist when K concentration below 1.07%. This was similar to the results reported by Grassi and Magnani^22^ and Tezara *et al.*^36^ in plants suffering from water stress. However, the investigation carried out by Galmés *et al.*^13^ revealed that B_L_ of *Hypericum balearicum* and *Phlomis italica* still remained zero under mild water stress even if the total limitation reached 20–30%. The present finding highlights that all photosynthetic limitations simultaneously occur when leaf is in a physiological K-deficiency state.

### Limitations vary with increasing K deficiency intensity

The leaf K concentration threshold value observed in this study was 1.07%, in consistent with the range of 0.5 to 2.0% reported by Leigh and Wyn Jones^37^. Quantitative limitation analysis gives insight into the contributions of different photosynthetic limitations, revealing that the B_L_ and MC_L_ accounted for the majority of total limitations in K-starved leaf margins and centers, respectively. This is mainly ascribed to the discrepancy of relative severity of K deficiency^5^,^6^. As has been stated in the previous studies that some irreversible damages, such as impaired ATP synthesis, depressed Rubisco activity, and cell damage occurred when the limiting *A*, for the most part, is attributed to B_L_^3^,^38^. Some of which were verified in the present study, such as degraded chloroplast, limited photoassimilate transportation (see Supplementary Fig. S2), and increased O_2_^.−^ generation rate under severe K deficiency. The obstacle of these physiological processes alleviated as K deficient stress mitigating, however, the role of MC_L_ on *A* began to stand out.

The relationship between relative limitations and leaf K concentration verified that, at a leaf K concentration of less than 1.07%, MC_L_ represented the main component of limitations, but B_L_ replaced it when leaf K concentration below 0.78%. This pattern, to a lesser extent, could be found in plants suffering from water stress which suggested that the variation of limitations depends on the stress intensity and duration^22^. Regrettably, the present study failed to reveal whether or not there is a critical concentration in the shifting process from S_L_ predominance to MC_L_ predominance. Further studies focusing on the photosynthetic limitations of rapeseed leaves subjected to a serial K gradient may help to elucidate this issue.

## Methods

### Study site and growth conditions

A field experiment was conducted in Wuxue county, Hubei province, central China (30° 06′46″N, 115° 36′9″E) during the 2013–2014 oilseed rape growing season. The mean temperature of the season was 13.8 °C, and the average temperature during winter (from December 2013 to February 2014) was 5.9 °C. The total precipitation during oilseed rape cropping season was 660.7 mm, with wintertide accounting for 26.1% of the total. The soil was a sandy loam with pH 5.3, organic matter 30.5 g kg^**−**1^, total N 1.7 g kg^**−**1^, NH_4_OAc-K 42.5 mg kg^**−**1^, Olsen-P 15.7 mg kg^**−**1^ and hot-water soluble B 0.78 mg kg^**−**1^ in the topsoil layer (0–20 cm). As stated by Zou, the soil belongs to a K-deficient type, which would cause yield reduction without K fertilizer addition^4^.

### Experimental design

A complete randomized block design was set up with two treatments and four replicates. The treatments were: (1) Sufficient K supply treatment (+K), with a K fertilizer recommendation rate of 120 kg K_2_O ha^–1^ which was tested and well-proved to ensure the optimal growth and yield formation of oilseed rape based on field experiments in this region^39^. (2) K deficiency treatment (–K), with no K fertilizer applied throughout the growing season.

To ensure that nutrients other than K did not limit plant K uptake, 180 kg N ha^**−**1^, 90 kg P_2_O_5_ ha^**−**1^, and 1.6 kg B ha^**−**1^ were applied for these two treatments. The N, P, K, B fertilizers used in the experiment consisted of urea (46% N), superphosphate (12% P_2_O_5_), potassium chloride (60% K_2_O), and borax (10.8% B). The N fertilizer was applied in three splits: 60% prior to transplanting, i.e., BBCH (Biologische Bundesantalt, Bundessortenamt and Chemische Industrie) 15–16^40^, 20% at the over-wintering stage (i.e., BBCH 29), and 20% at the initiation of stem elongation (i.e., BBCH 30). Besides, all the P, K, B fertilizers were applied as basal fertilizers. The experimental field was plowed and leveled with a rotary tiller, and basal fertilizers were incorporated during the process. The plot measured 20 m^2^ with a length of 10 m and a width of 2 m.

The oilseed rape cultivar was Zhongshuang 11, supplied by Oil Crops Research Institute of the Chinese Academy of Agricultural Sciences. Rapeseeds were sown in prepared seedbeds on 16 September 2013, and then, on 22 October, about 36 d after sowing, oilseed-rape seedlings with five to six leaves (i.e., BBCH 15–16, 3–4 g dry weight plant^**−**1^) were uniformly selected and transplanted by hand in double rows spaced approximately 0.3 m apart with 0.2–0.3 m between plants, corresponding to 112 500 plants ha^–1^. The oilseed rape was grown under rain-fed conditions. Meanwhile, weeds, pests and disease stresses were controlled by spray herbicides, insecticide and fungicide so that no obvious weeds, insect pests, and diseases infestation occurred during cropping season.

### Plant and leaf tagging

There was an obvious phenotypic difference in plants between the –K and +K treatments 60 d after transplanting. The discrepancy was highlighted in the fifth to ninth fully expanded leaves (with a total average of 9 fully expanded leaves (i.e., BBCH 19) in both treatments) from apex downwards, specifically, obvious etiolation symptoms around the periphery in K-deficient leaves and asymptomatic leaves in the +K treatment. For each treatment, 24 fifth fully expanded leaves and six uniform plants were tagged in each of the four replicate plots for destructive and non-destructive analysis described later in the methods.

### Leaf gas exchange and fluorescence measurements

**Figure 4:**
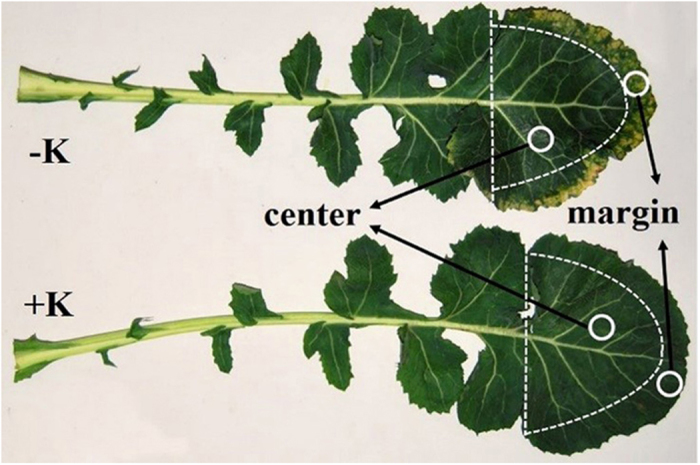
Leaves under the –K and +K treatments. The vertical dash lines divide leaf into two parts, the leaf apexes are separated into leaf margins and leaf centers by half-elliptic lines. White circles indicate the gas exchange measuring positions.

Leaf gas exchange and chlorophyll fluorescence were measured simultaneously at either leaf margins (Fig. 4, about 2 cm of leaf surface from the margin) or leaf centers (the rest part between half-elliptic and vertical dashed lines), using a portable, open circuit, infrared gas analysis system (Li-6400, Li-Cor Inc., Lincoln, NE, USA) equipped with an integrated leaf chamber fluorometer (Li-6400–40). Measurements were performed on at least four randomly selected leaves of both treatments in the late morning (11:00–12:30) under a light-saturating photosynthetic photon flux density (PPFD) of 1200 μmol m^**−**2^s^**−**1^ (with 90% red light and 10% blue light). CO_2_ concentration in the leaf chamber (*C*_a_) was set at 400 μmol mol^**−**1^ air, leaf temperature was controlled at 25 ± 0.2 °C, relative humidity was between 50 and 60%, and the flow rate was 500 μmol s^**−**1^. In addition to net photosynthesis (*A*), stomatal conductance to water vapour (*g*_s_) and intercellular CO_2_ concentration (*C*_i_), the incorporated fluorometer allowed determination the steady-state fluorescence yield (*F*_s_) under actinic light and maximum fluorescence (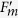) during light-saturating pulse (0.8 s) of approx. 8000 μmol m^–2^ s^–1^. The relative *A*, *g*_s_ and *g*_m_ values were the relative proportion of measured values over the mean values of K-sufficient leaf centers.

*A*/*C*_i_ curves were measured on the two positions that had been previously acclimated to saturating light conditions for 20 min. The CO_2_ concentration (*C*_a_) in the gas exchange chamber was reduced stepwise from 400 to 300, 250, 200, 150, 100, 50 μmolCO_2_ mol^**−**1^, and then increased from 50 to 400, 600, 800, 1000, 1200, 1500, 1800 μmol CO_2_ mol^**−**1^ at a constant PPFD of 1200 μmol m^**−**2^s^**−**1^ at 25 ± 0.2 °C, and 50–60% relative humidity. In all cases, the parameters were recorded after the gas exchange rate stabilized at the given *C*_a_. At least four leaves were performed in each treatment.

The actual photochemical efficiency of photosystem II (Φ_PSII_) was then determined as follows^41^:


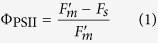


The electron transport rate (*J*) can be calculated as:


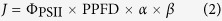


Where *α* is the leaf absorptance, and *β* is the fraction of light distributed to PSII. As routinely assumed, α was taken as 0.85^42^,^43^ and β was taken as 0.5^44^,^45^. A sensitivity analysis of *J* biases resulting from rough assumption of α and β on *g*_m_ variations was also conducted (See Supplementary Table S5).

Mesophyll conductance was estimated according to Harley *et al.* from combined gas exchange and chlorophyll fluorescence measurements^46^.


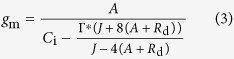


where *A*, *C*_i_ and *J* were determined as previously described for each treatment, mitochondrial respiration rate in the light (*R*_d_) and the intercellular CO_2_ compensation point (*C*_i_^*^) were measured by Laisk method, as described by Brooks and Farquhar^47^. Briefly, the *A*/*C*_i_ curves generated with PPFD values of 75, 150, 500 μmol m^**−**2^s^**−**1^, respectively, with each having five different *C*_a_ in chamber (i.e. 50, 80, 100, 120 and 150 μmol CO_2_ mol^**−**1^). A linear regression was then fitted to each *A*/*C*_i_ curve. The *x*-axis and *y*-axis of intersection point of three *A*/*C*_i_ curves were defined as *C*_i_^*^ and *R*_d_^48^. The Γ^*^ is the chloroplastic CO_2_ photocompensation point calculated from *C*_i_^*^ and *R*_d_ as:


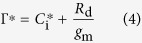


For each data point generated, we checked whether it met the range of 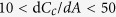
46. The CO_2_ concentration in the chloroplast stroma (*C*_c_) was calculated as:


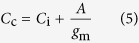


Therefore, *A*-*C*_i_ curves were converted into *A*-*C*_c_ curves. On the basis of *C*_c_, the maximum rate of Rubisco-catalysed carboxylation (*V*_*c,*max_), and the maximum rate of electron transport (*J*_max_) as defined by Farquhar *et al.*^49^, were calculated^11^,^50^.

Since variable *J* method is sensitive to many sources of errors, e.g. (1) Γ^*^ and *R*_d_ biases; (2) a wrong assumption of *p*_1_ and *p*_2_; (3) biases in the measurements of *C*_i_, *A*, and *J*, a sensitivity analysis would be great values to improve the confidence in *g*_m_ estimates and following limitation calculations^51^. Following the method of Harley *et al.*^46^, we used actual Γ*, *R*_d_ and *J* values calculated in this study and a deviation from the measured values to analyze the effects of Γ^*^, *R*_d_ and *J* on *g*_m_ estimates (see Supplementary Table S1, S3, S5). RuBP regeneration can be limited by either insufficient NADPH or ATP, according to Farquhar model, *A* and *J* can be linked as follows:


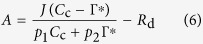


For insufficient NADPH, *p*_1_ = 4 and *p*_2_ = 8; for insufficient ATP, *p*_1_ = 4.5 and *p*_2_ = 10.5 or *p*_1_ = 4 and *p*_2_ = 9.33. Finally, the sensitivity analysis for photosynthetic limitations was conducted basing on these calculated *g*_m_ values (see Supplementary Table S2, S4, S6). The analysis showed that the *g*_m_ was significantly affected by varying Γ^*^ and *R*_d_ (see Supplementary Table S1), *p*_1_ and *p*_2_ inputs (see Supplementary Table S3) and *J* biases (see Supplementary Table S5). However, the *g*_m_ variation derived from Γ^*^, *R*_d_, *J*, *p*_1_ and *p*_2_ biases, did not cause profound effects on photosynthetic limitations (see Supplementary Table S2, S4, S6). In addition, *g*_m_ appears to be strikingly affected by *C*_i_^12^,^14^,^51^, nevertheless, the similar *C*_i_ in different treatments and positions here seems to have no impact on *g*_m_ (Table 3). Therefore, the results obtained was unlikely to be altered by these methodological artifacts.

### Plant dry matter, leaf area and dry matter

Six tagged leaves and six tagged plants in each plot were used to determine the individual leaf area, dry matter, and total dry matter. Each leaf was digitally scanned using an Epson ES-1200C scanner (Epson, Long Beach, CA, USA), and the area determined using ImageJ software (National Institutes of Health, Bethesda, Maryland, USA)^1^. Individual leaf dry matter and total dry matter were weighed after oven drying at 65 °C for 48 h.

### Biochemical analysis

Twelve tagged leaves per plot were picked immediately after the determination of photosynthesis. They were divided into two parts along vertical dashed lines (Fig. 4), followed by dissecting the leaf apexes into leaf margins and leaf centers, and removing all the veins. A portion of segments were immersed in liquid N and then stored at −78 °C, and the rest were used for leaf K concentration determination. There were four replications for biochemical determinations.

Leaf segments (2 g) were oven dried at 65 °C for 48 h. After that, about 0.15 g dried leaves were milled and digested with H_2_SO_4_-H_2_O_2_ as described by Thomas *et al.*^52^, and K concentration in digestion solution was measured by a flame photometer (M-410, Cole-Parmer, Chicago, IL, USA).

The Rubisco extracts were prepared according to Weng *et al.* with minor modifications^3^. Briefly, leave segments (0.2 g) were ground to a powder using a chilled mortar and pestle with liquid N_2_ and a small amount of quarzsand, followed by homogenization with 4 mL pre-cooled extraction buffer containing 50 mM Tris-HCl (pH 7.5), 1 mM EDTA,10 mM MgCl_2_, 12.5% (v/v) glycerol, 10 mM (v/v) β-mercaptoethanol and 1% (w/v) PVP-40 (soluble PVP) at 0–4 °C. The homogenate was centrifuged for 15 min at 15 000 *g* at 4 °C, and then the supernatant was immediately used to determine the activity of ribulose-1, 5-bisphosphate carboxylase/oxygenase (Rubisco, EC 4.1.1.39) by an enzyme-linked immunosorbent assay method with a RuBPcase ELISA kit (CK-E91697P, Shanghai jijin Chemistry and Technology Co., Ltd, China) according to the manufacturer’s instructions. The chlorophyll concentration was determined according to the method of Huang *et al.*^53^.

Superoxide radical O_2_^.−^ production rate was measured by monitoring the nitrite formation from hydroxylamine in the presence of O_2_^.−^ according to Elstner and Heupel^54^. A 0.5 g aliquot of leaf margins and centers was ground and homogenized in 5 mL of 65 mM pre-cooled phosphate buffer (pH 7.8), followed by centrifuging the homogenate at 10,000 g for 15 min at 4 °C and mixing 0.5 mL of the supernatant with phosphate buffer (0.5 mL) and 0.1 mL of 10 mM hydroxylamine hydrochloride. This mixture was incubated at 25 °C for 20 min, followed by the addition of 1 mL of 58 mM sulfanilic acid and 1 mL of α-naphthylamine, and then another 20 min incubation at 25 °C. The as-prepared solution was shaken with equal volume of ether, followed by centrifuging the mixture at 10,000 g for 3 min and measuring the absorbance of the pink water phase at 530 nm. The activity of POD (EC 1.11.1.7) was determined using the guaiacol oxidation method^55^.

### Anatomical analysis

Another six tagged leaves per treatment were collected, and removed all the veins for anatomical analysis. The stomatal size and frequency were measured in six sub-samples either for leaf margin or center. The materials were prepared as described by Meng *et al.*^56^. Briefly, leaf samples (about 1cm in length and 1 cm in width) were fixed in 2.5% glutaraldehyde (v/v) at 4 °C for 2 h, and washed twice in 0.1 M phosphate buffer (pH 6.8). Next, they were sequentially dehydrated in ethanol (30%, 50%, 70%, 80%, 90%, 95%, and 100%) for 10 min at each gradient concentration, with 100% ethanol repeated twice. After further drying and spraying with gold, the as-treated leaf samples were observed and photographed with a scanning electron microscope (JSM-5310LV, Jeol Co, Tokyo, Japan). Images were taken of the lower leaf surface for five microscope fields per sub-sample at a magnification of ×500. The number of stomata was counted in each field (a total of 20 measurements of stomatal frequency for each position) as described by Battie-Laclau *et al.*^1^, and the stomatal frequency was calculated by dividing the stomatal count by the area of the field of view^57^. Moreover, the length and width of ten stomata selected at random were measured in each field. Assuming the stomatal pore as an ellipse, the total stomatal pore area was calculated (stomatal frequency × π × 0.25 × stomatal length × stomatal width).

Leaf segments (1–2 mm^2^) were cut from each part and fixed with 2.5% glutaraldehyde (v/v) in 0.1 M phosphate buffer (pH 7.4) for 4 h, followed by washing twice in the same buffer for 30 min and postfixing with 2% osmium tetroxide for 4 h at 4 °C. Next, the samples were dehydrated with an ethanol series (10–100%) and in propylene oxide, followed by embedding them in Epon 812 resin.

For the light microscope observation, they were cut into 1 μm transverse sections by LKB-5 ultramicrotome 359 (LKB Co., Ltd., Uppsala, Sweden), and stained with 0.5% toluidine blue. Micrographs were captured at a magnification of ×400 with a Nikon Eclipse E600 microscope equipped with a Nikon 5 MP digital microscope camera DS-Fi1 (Nikon Corporation, Kyoto, Japan). There were four samples per treatment. For each samples, three cross-sections were chosen to measure their thickness (*T*_leaf_), mesophyll cell wall surface area exposed to intercellular airspace per leaf area (*S*_m_/*S*), and surface area of chloroplasts exposed to intercellular airspaces (*S*_c_/*S*) according to Tosens *et al.* (2012)^32^.


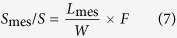



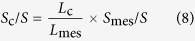


Where *L*_mes_ and *L*_c_ are the length of mesophyll cell wall exposing to intercellular air space and chloroplast surface area touching the intercellular air space. *W* is the width of measured cross-section. *F* is the curvature correction factor which was obtained as the weight average of palisade and spongy mesophyll.

For the ultrastructural observations, ultrathin sections (90 nm) were examined with a transmission electron 360 microscope (H-7650, Hitachi, Japan) after staining with 2.0% uranyl acetate (w/v) and lead citrate. Cell wall thickness (*T*_cell-wall_), chloroplast length (*L*_chl_) and thickness (*T*_chl_) were measured from at least 30 chloroplasts. Chloroplasts were assumed as ellipsoids, and chloroplast surface area (*S*_chl_) and volume (*V*_chl_) were calculated according Cesaro formula^58^:


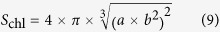



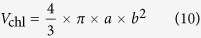


where 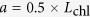; 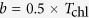. Distance of chloroplast from cell wall (*D*_chl-cw_) was determined according to Tomás *et al.*^23^.

### Quantitative limitation analysis

The limitations (stomatal limitations, S_L_; mesophyll conductance limitations, MC_L_; biochemical limitations, B_L_) imposed by K deficiency on photosynthesis were investigated by analyzing the leaf margins and centers under two treatments using the quantitative limitation analysis method proposed by Grassi and Magnai^22^. Relative changes in light-saturated assimilation is expressed in terms of relative changes in stomatal, mesophyll conductance, and biochemical capacity as Equation (11).


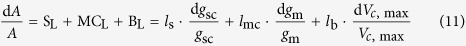


where *l*_s_, *l*_mc_, and *l*_b_ are the corresponding relative limitations calculated as Eqns from (12) to (14), *g*_sc_ is stomatal conductance to CO_2_ (*g*_s_/1.6), and *V*_c,max_ is maximum rate of carboxylation estimated from *A*-*C*_i_ curve.


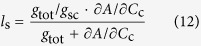



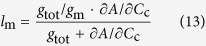



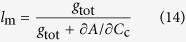


where *g*_tot_ is the total conductance to CO_2_ from leaf surface to carboxylation sites determined as Equation (15). By following Tomás *et al.*^23^, 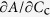 was calculated as slope of *A*-*C*_c_ response curves over a *C*_c_ range of 50–100 μmol mol^–1^.


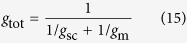


Then the relative change of *A*, *g*_sc_, *g*_m_ and *V*_c,max_ in Equation (11) can be approximated by Chen *et al.*^59^.


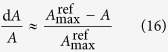



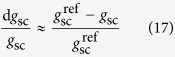



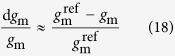



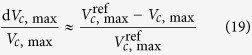


where 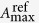, 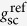, 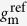 and 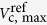 are the reference values of net CO_2_ assimilation rate, stomatal conductance and mesophyll conductance, and the rate of carboxylation, defined as maximun value measured under light saturation. In the original reference, the authors used the maximum value of seasonal *A*_max_ under light-saturated conditions as a reference to assess the photosynthetic limitations of *A* for each determination. In the current study, the maximum *A* was generally reached, concomitantly with *g*_s_, *g*_m_ and *V*_c,max_ in the leaf centers with the +K treatment, and the mean values of the +K treatments was thus used as a reference, i.e., there was no limitation present in the leaf centers under the +K treatment. Whenever one of the three parameters was higher in either one of the rest treatment than that of the reference, its corresponding limitation was set to zero. In this way, the limitations in the leaf margins and centers under the –K treatment could be quantified. Finally, non-stomatal limitations were defined as the sum of mesophyll conductance limitations and biochemical limitations (NS_L_ = MC_L_ + B_L_).

### Statistical analysis

One-way analysis of variance (*ANOVA*) was caiculated usig SPSS 18.0 software (SPSS, Chicago, IL, USA). The mean values were compared using the least significant difference (LSD) test (*P* < 0.05). Graphics and regression analysis were performed using the OriginPro 8.5 software (OriginLab Corporation, Northampton, MA, USA).

## Additional Information

**How to cite this article**: Lu, Z. *et al.* Differences on photosynthetic limitations between leaf margins and leaf centers under potassium deficiency for *Brassica napus* L. *Sci. Rep.* **6**, 21725; doi: 10.1038/srep21725 (2016).

## Supplementary Material

Supplementary Information

## Figures and Tables

**Table 1:** Effects of K deficiency on plant dry matter, leaf dry matter, leaf area, leaf K concentration, and net CO_2_ assimilation rate (*A*) for the two positions in the fifth fully expanded leaves.

Treatment	Position	Total dry matter (g)	Individual leaf dry matter (g)	Leaf area (cm^2^)	K concentration (% of dry weight)	*A*(μmol CO_2_ m^−2^ s^−1^)
–K	margin	21.1 ± 2.6b^1^	2.31 ± 0.03b	320 ± 4b	0.65 ± 0.02b^2^	9.5 ± 0.2b
	center				0.98 ± 0.05a	16.5 ± 0.6a
+K	margin	30.1 ± 1.1a	2.97 ± 0.16a	391 ± 8a	1.32 ± 0.06b*^3^	17.0 ± 0.3a*
	center				1.66 ± 0.06a*	17.3 ± 0.7a

Values are mean ± SE of six replications for total dry matter, individual leaf dry matter and leaf area, and of four replications for K concentration and *A*.

^1^Different letters in the same column of total dry matter, individual leaf dry matter and leaf area indicate significant differences between treatments (*P* ≤ 0.05).

^2^Different letters in the same column of K concentration and *A* indicate significant differences between positions (*P* ≤ 0.05).

^3^*shows significant differences between the two K treatment in same position (*P* ≤ 0.05).

**Table 2:** Effects of K deficiency on stomatal conductance (g_s_), intercellular CO_2_ concentrations (C_i_), stomatal frequency, length, and width, single stomatal pore area and total stomatal pore area of the two positions in the lower epidermis of the fifth fully expanded leaves.

Treatment	Position	*g*_s_(mol H_2_O m^−2^ s^−1^)	*C*_i_(μmol CO_2_mol^−1^)	Stomatal frequency (no. mm^−2^)	Stomatal length (μm)	Stomatal width (μm)	Single stomatal pore area (μm^2^)	Total stomatal pore area (10^−3^ mm^2^ mm^−2^)
−K	margin	0.142 ± 0.006b^1^	257 ± 6a	334.1 ± 7.0a	9.11 ± 0.07a	2.91 ± 0.02b	20.87 ± 0.15b	6.97 ± 0.05b
	center	0.231 ± 0.012a	240 ± 5a	334.7 ± 8.5a	9.94 ± 0.04a	3.49 ± 0.03a	27.30 ± 0.12a	9.14 ± 0.04a
+K	margin	0.223 ± 0.017a*^2^	238 ± 7a	335.7 ± 14.4a	10.01 ± 0.05a	3.68 ± 0.02a*	28.90 ± 0.14a*	9.70 ± 0.05a*
	center	0.238 ± 0.011a	231 ± 3a	338.5 ± 7.9a	10.66 ± 0.04a	3.54 ± 0.02a	29.64 ± 0.15a	10.03 ± 0.05a*

Images were taken at a magnification of ×500 with a scanning electron microscope. Values are mean ± SE of four replications for *g*_s_, *C*_i_, of 20 replications for stomatal frequencies, and of 300 replications for stomatal lengths, stomatal widths, stomatal pore areas and total stomatal pore areas.

^1^Different letters in the same column at a given treatment indicate significant differences between positions (*P* ≤ 0.05).

^2^*shows significant differences between the two K treatment in same position (*P* ≤ 0.05).

**Table 3:** Effects of K deficiency on mesophyll conductance (g_m_), chloroplastic CO_2_ concentrations (*C*_c_), intercellular CO_2_ compensation point (*C*_i_^*^), mitochondrial respiration rate in the light (*R*_d_), and chloroplastic CO_2_ compensation point (Γ*) in the two positions of the fifth fully expanded leaves.

Treatment	Position	*g*_m_(mol CO_2_m^−2^s^−1^)	*C*_c_(μmol CO_2_mol^−1^)	*C*_i_^*^ (μmol CO_2_mol^−1^)	*R*_d_(μmol CO_2_ m^−2^ s^−1^)	Γ* (μmol CO_2_mol^−1^)
−K	margin	0.084 ± 0.002b^1^	139 ± 3a	36.2 ± 0.8a	0.95 ± 0.03a	46.5 ± 1.6a
	center	0.151 ± 0.008a	132 ± 5b	35.4 ± 0.9a	0.92 ± 0.03a	41.7 ± 2.1a
+K	margin	0.163 ± 0.003a*^2^	127 ± 2a*	33.5 ± 1.4a	0.89 ± 0.05a	40.1 ± 1.5a*
	center	0.174 ± 0.018a	130 ± 7a	32.4 ± 1.7a	0.87 ± 0.04a	39.4 ± 2.0a

*C*_i_^*^ and *R*_d_ were measured by Laisk method, Γ* was calculated according to the equation Γ* = *C*_i_^*^ + *R*_d_/*g*_m_. Values are mean ± SE of four replications for *g*_m_, *C*_c_, and *C*_i_-*C*_c_, of three replications for *C*_i_^*^, *R*_d_ and Γ*.

^1^Different letters in the same column at a given treatment indicate significant differences between positions (*P* ≤ 0.05).

^2^*shows significant differences between the two K treatment in same position (*P* ≤ 0.05).

**Table 4:** Effects of K deficiency on the maximum rate of electron transport (*J*_max_), maximum rate of carboxylation (*V*_c,max_), ratio between *J*_max_ and *V*_c,max_ (*J*_max_/*V*_c,max_) estimated from *A*-*C*_c_ curves, chlorophyll concentration, Rubisco activity, O_2_^.−^ generation rate, and POD activity in the two positions of the fifth fully expanded leaves.

Treatment	Position	*J*_max_(μmol m^−2^s^−1^)	*V*_c,max_(μmol m^−2^s^−1^)	*J*_max_/*V*_c,max_	Chlorophyll (g m^−2^)	Rubisco activity (U g^−1^ FW)	O_2_^.−^ generation rate (nmol g^−1^ FW min^−1^)	POD activity (U g^−1^ FW min^−1^)
−K	margin	114.5 ± 9b^1^	70.9 ± 4.8b	1.61 ± 0.12a	0.42 ± 0.03b	0.29 ± 0.02b	8.77 ± 0.35a	2868 ± 176a
	center	166.2 ± 10a	113.9 ± 4.5a	1.46 ± 0.03b	0.54 ± 0.02a	0.36 ± 0.01a	6.97 ± 0.15b	2511 ± 74b
+ K	margin	170.6 ± 12a*	120.5 ± 5.9a*	1.42 ± 0.06a*	0.61 ± 0.05a*	0.33 ± 0.01a*	7.18 ± 0.22a*	2275 ± 111a*
	center	167.5 ± 9a	118.0 ± 5.6a	1.42 ± 0.13a	0.64 ± 0.04a*	0.35 ± 0.01a	6.61 ± 0.33a	2085 ± 148a*

Values are mean ± SE of four replications.

^1^Different letters in the same column at a given treatment indicate significant differences between positions (*P* ≤ 0.05).

^2^*shows significant differences between the two K treatment in same position (*P* ≤ 0.05).

**Table 5:** Effects of K deficiency on leaf thickness (*T*_leaf_), mesophyll cell wall thickness (*T*_cell-wall_), mesophyll cell wall surface area exposed to intercellular airspace per leaf area (*S*_m_/*S*), and surface area of chloroplasts exposed to intercellular airspaces (*S*_c_/*S*) in the two positions of the fifth fully expanded leaves.

Treatment	Position	*T*_leaf_(μm)	*T*_cell-wall_(μm)	*S*_m_/*S*(m^2^ m^−2^)	*S*_c_/*S*(m^2^ m^−2^)
−K	margin	305 ± 27.7a	0.167 ± 0.017a	12.9 ± 1.8b	8.6 ± 1.1b
	center	310 ± 19.2a	0.170 ± 0.033a	17.1 ± 2.4a	12.8 ± 0.9a
+ K	margin	309 ± 31.3a	0.172 ± 0.049a	17.4 ± 3.3a*^2^	13.1 ± 1.6a*
	center	312 ± 22.0a	0.176 ± 0.045a	18.2 ± 2.6a	13.5 ± 2.1a

Data are mean ± SE of eight replications for *S*_m_/*S* and *S*_c_/*S*, at least thirty replications for *T*_leaf_ and *T*_cell-wall_.

^1^Different letters in the same column at a given treatment indicate significant differences between positions (*p* ≤ 0.05).

^2^* shows significant differences between the two K treatment in same position (*p* ≤ 0.05).

## References

[b1] Battie-Laclau P. *et al.* Photosynthetic and anatomical responses of *Eucalyptus grandis* leaves to potassium and sodium supply in a field experiment. Plant cell environ. 37, 70–81 (2014). 23663049 10.1111/pce.12131

[b2] Erel R. *et al.* Modification of non-stomatal limitation and photoprotection due to K and Na nutrition of olive trees. J. Plant physiol. 177, 1–10 (2015). 25659331 10.1016/j.jplph.2015.01.005

[b3] Weng X. Y., Zheng C. J., Xu H. X. & Sun J. Y. Characteristics of photosynthesis and functions of the water-water cycle in rice (*Oryza sativa*) leaves in response to potassium deficiency. Physiol. plantarum 131, 614–621 (2007). 10.1111/j.1399-3054.2007.00978.x 18251852

[b4] Zou J., Lu J. W., Li Y. S. & Li X. K. Regional evaluation of winter rapeseed response to K fertilization, K use efficiency, and critical level of soil K in the Yangtze River Valley. Sci. Agric. Sin. 10, 911–920 (2011).

[b5] Bednarz C., Oosterhuis D. & Evans R. Leaf photosynthesis and carbon isotope discrimination of cotton in response to potassium deficiency. Environ. Exp. Bot. 39, 131–139 (1998).

[b6] Jin S. H. *et al.* Effects of potassium supply on limitations of photosynthesis by mesophyll diffusion conductance in *Carya cathayensis*. Tree physiol. 31, 1142–1151 (2011). 21990026 10.1093/treephys/tpr095

[b7] Flexas J., Ribas-Carbó M., Diaz-Espejo A., Galmés J. & Medrano H. Mesophyll conductance to CO_2_: current knowledge and future prospects. Plant Cell Environ. 31, 602–621 (2008). 17996013 10.1111/j.1365-3040.2007.01757.x

[b8] Lebaudy A. *et al.* Plant adaptation to fluctuating environment and biomass production are strongly dependent on guard cell potassium channels. P. Natl. Acad. Sci. USA 105, 5271–5276 (2008). 10.1073/pnas.0709732105 PMC2278230 18367672

[b9] Andrés Z. *et al.* Control of vacuolar dynamics and regulation of stomatal aperture by tonoplast potassium uptake. P. Natl. Acad. Sci. USA 111, 1806–1814 (2014). 10.1073/pnas.1320421111 PMC4035970 24733919

[b10] Jordan-Meille L. & Pellerin S. Shoot and root growth of hydroponic maize (*Zea mays* L.) as influenced by K deficiency. Plant Soil 304, 157–168 (2008).

[b11] Bernacchi C. J., Portis A. R., Nakano H., von Caemmerer S. & Long S. P. Temperature response of mesophyll conductance. Implications for the determination of Rubisco enzyme kinetics and for limitations to photosynthesis *in vivo*. Plant Physiol. 130, 1992–1998 (2002). 12481082 10.1104/pp.008250 PMC166710

[b12] Flexas J. *et al.* Rapid variations of mesophyll conductance in response to changes in CO_2_ concentration around leaves. Plant Cell Environ. 30, 1284–1298 (2007). 17727418 10.1111/j.1365-3040.2007.01700.x

[b13] Galmés J., Medrano H. & Flexas J. Photosynthetic limitations in response to water stress and recovery in Mediterranean plants with different growth forms. New Phytol. 175, 81–93 (2007). 17547669 10.1111/j.1469-8137.2007.02087.x

[b14] Xiong D. L. *et al.* Rapid responses of mesophyll conductance to changes of CO_2_ concentration, temperature and irradiance are affected by N supplements in rice. Plant Cell Environ. doi: 10.1111/pce.12558 (2015). 25923314

[b15] Kaldenhoff R. Mechanisms underlying CO_2_ diffusion in leaves. Curr. Opin. Plant Biol. 15, 276–281 (2012). 22300606 10.1016/j.pbi.2012.01.011

[b16] Kanai S. *et al.* Potassium deficiency affects water status and photosynthetic rate of the vegetative sink in green house tomato prior to its effects on source activity. Plant sci. 180, 368–374 (2011). 21421382 10.1016/j.plantsci.2010.10.011

[b17] Wang N. *et al.* Genotypic variations in photosynthetic and physiological adjustment to potassium deficiency in cotton (*Gossypium hirsutum*). J. Photochem. Photobiol. B: Biol. 110, 1–8 (2012). 10.1016/j.jphotobiol.2012.02.002 22387141

[b18] Niinemets Ü. *et al.* Do we underestimate the importance of leaf size in plant economics? Disproportional scaling of support costs within the spectrum of leaf physiognomy. Ann. Bot. 100, 283–303 (2007). 17586597 10.1093/aob/mcm107 PMC2735320

[b19] Cakmak I. The role of potassium in alleviating detrimental effects of abiotic stresses in plants. J. Plant Nutr. Soil Sci. 168, 521–530 (2005).

[b20] Paul M. J. & Pellny T. K. Carbon metabolite feedback regulation of leaf photosynthesis and development. J. Exp. Bot. 54, 539–547 (2003). 12508065 10.1093/jxb/erg052

[b21] Araya T., Noguchi K. & Terashima I. Effects of carbohydrate accumulation on photosynthesis differ between sink and source leaves of *Phaseolus vulgaris* L. Plant cell physiol. 47, 644–652 (2006). 16540483 10.1093/pcp/pcj033

[b22] Grassi G. & Magnani F. Stomatal, mesophyll conductance and biochemical limitations to photosynthesis as affected by drought and leaf ontogeny in ash and oak trees. Plant Cell Environ. 28, 834–849 (2005).

[b23] Tomás M. *et al.* Importance of leaf anatomy in determining mesophyll diffusion conductance to CO_2_ across species: quantitative limitations and scaling up by models. J. Exp. Bot. 64, 2269–2281 (2013). 23564954 10.1093/jxb/ert086 PMC3654418

[b24] Gago J. *et al.* Photosynthesis limitations in three fern species. Physiol. plantarum 149, 599–611 (2013). 10.1111/ppl.12073 23692357

[b25] Muir C. D., Hangarter R. P., Moyle L. C. & Davis P. A. Morphological and anatomical determinants of mesophyll conductance in wild relatives of tomato (*Solanum* sect. *Lycopersicon*, sect. *Lycopersicoides*; Solanaceae). Plant cell environ. 37, 1415–1426 (2014). 24279358 10.1111/pce.12245

[b26] Ren T. *et al.* Potassium-fertilizer management in winter oilseed-rape production in China. J Plant Nutr. Soil Sci. 176, 429–440 (2013).

[b27] Gierth M. & Mäser P. Potassium transporters in plants–involvement in K^+^ acquisition, redistribution and homeostasis. FEBS lett. 581, 2348–2356 (2007). 17397836 10.1016/j.febslet.2007.03.035

[b28] Gonzalez N., Vanhaeren H. & Inzé D. Leaf size control: complex coordination of cell division and expansion. Trends Plant Sci. 17, 332–340 (2012). 22401845 10.1016/j.tplants.2012.02.003

[b29] Zörb C., Senbayram M. & Peiter E. Potassium in agriculture-status and perspectives. J. Plant Physiol. 171, 656–669 (2014). 24140002 10.1016/j.jplph.2013.08.008

[b30] Fanaei H., Galavi M., Kafi M. & Bonjar A. G. Amelioration of water stress by potassium fertilizer in two oilseed species. Int. J. Plant Prod. 3, 41–54 (2009).

[b31] Peiter E. The plant vacuole: emitter and receiver of calcium signals. Cell Calcium 50, 120–128 (2011). 21376393 10.1016/j.ceca.2011.02.002

[b32] Tosens T., Niinemets Ü., Westoby M. & Wright I. J. Anatomical basis of variation in mesophyll resistance in eastern Australian sclerophylls: news of a long and winding path. J. Exp. Bot. 63, 5105–5119 (2012). 22888123 10.1093/jxb/ers171 PMC3430992

[b33] Li Y. *et al.* Does chloroplast size influence photosynthetic nitrogen use efficiency? PloS ONE 8, e62036 doi: 10.1371/journal.pone.0062036 (2013). 23620801 PMC3631174

[b34] Xiong D. L. *et al.* SPAD-based leaf nitrogen estimation is impacted by environmental factors and crop leaf characteristics. Scientific Reports 5, 13389 doi: 10.1038/srep13389 (2015). 26303807 PMC4548214

[b35] Evans J. R., von Caemmerer S., Setchell B. A. & Hudson G. S. The relationship between CO_2_ transfer conductance and leaf anatomy in transgenic tobacco with a reduced content of Rubisco. Aust. J. Plant Physiol. 21, 475–495 (1994).

[b36] Tezara W., Mitchell V., Driscoll S. & Lawlor D. Effects of water deficit and its interaction with CO_2_ supply on the biochemistry and physiology of photosynthesis in sunflower. J. Exp. Bot. 53, 1781–1791 (2002). 12147728 10.1093/jxb/erf021

[b37] Leigh R. A. & Wyn Jones R. G. A hypothesis relating critical potassium concentrations for growth to the distribution and functions of this ion in the plant cell. New Phytol. 97, 1–13 (1984).

[b38] Flexas J., Bota J., Loreto F., Cornic G. & Sharkey T. D. Diffusive and metabolic limitations to photosynthesis under drought and salinity in C_3_ Plants. Plant Biology 6, 269–279 (2004). 15143435 10.1055/s-2004-820867

[b39] Li Y. S. *et al.* Study on response to potassium (K) application and recommendation of optimal K rates for rapeseed in Hubei. Chin. J. Oil Crop Sci. 30, 469–475 (2008).

[b40] Lancashire P. D. *et al.* A uniform decimal code for growth stages of crops and weeds. Ann. Appl. Biol. 119, 561–601 (1991).

[b41] Genty B., Briantais J. M. & Baker N. R. The relationship between the quantum yield of photosynthetic electron transport and quenching of chlorophyll fluorescence. BBA-Gen. Subjects 990, 87–92 (1989).

[b42] Li Y., Gao Y. X., Xu X. M., Shen Q. R. & Guo S. W. Light-saturated photosynthetic rate in high-nitrogen rice (*Oryza sativa* L.) leaves is related to chloroplastic CO_2_ concentration. J. Exp. Bot. 60, 2351–2360 (2009). 19395387 10.1093/jxb/erp127

[b43] Manter D. K. & Kerrigan J. *A/C*_i_ curve analysis across a range of woody plant species: influence of regression analysis parameters and mesophyll conductance. J. Exp. Bot. 55, 2581–2588 (2004). 15501912 10.1093/jxb/erh260

[b44] Albertsson P.-A. A quantitative model of the domain structure of the photosynthetic membrane. Trends Plant Sci. 6, 349–354 (2001). 11495787 10.1016/s1360-1385(01)02021-0

[b45] Warren C. R. The photosynthetic limitation posed by internal conductance to CO_2_ movement is increased by nutrient supply. J. Exp. Bot. 55, 2313–2321 (2004). 15310814 10.1093/jxb/erh239

[b46] Harley P. C., Loreto F., Di Marco G. & Sharkey T. D. Theoretical considerations when estimating the mesophyll conductance to CO_2_ flux by analysis of the response of photosynthesis to CO_2_. Plant Physiol. 98, 1429–1436 (1992). 16668811 10.1104/pp.98.4.1429 PMC1080368

[b47] Brooks A. & Farquhar G. D. Effect of temperature on the CO_2_/O_2_ specificity of ribulose-1, 5-bisphosphate carboxylase/oxygenase and the rate of respiration in the light. Planta 165, 397–406 (1985). 24241146 10.1007/BF00392238

[b48] von Caemmerer S., Evans J., Hudson G. & Andrews T. J. The kinetics of ribulose-1,5-bisphosphate carboxylase/oxygenase *in vivo* inferred from measurements of photosynthesis in leaves of transgenic tobacco. Planta 195, 88–97 (1994).

[b49] Farquhar G. D., von Caemmerer S. V. & Berry J. A. A biochemical model of photosynthetic CO_2_ assimilation in leaves of C_3_ species. Planta 149, 78–90 (1980). 24306196 10.1007/BF00386231

[b50] Sharkey T. D., Bernacchi C. J., Farquhar G. D. & Singsaas E. L. Fitting photosynthetic carbon dioxide response curves for C_3_ leaves. Plant Cell Environ. 30, 1035–1040 (2007). 17661745 10.1111/j.1365-3040.2007.01710.x

[b51] Gu L. H. & Sun Y. Artefactual responses of mesophyll conductance to CO_2_ and irradiance estimated with the variable *J* and online isotope discrimination methods. Plant Cell Environ. 37, 1231–1249 (2014). 24237289 10.1111/pce.12232

[b52] Thomas R. L., Sheard R. W. & Moyer J. R. Comparison of conventional and automated procedures for nitrogen, phosphorus, and potassium analysis of plant material using a single digestion. Agron. J. 59, 240–243 (1967).

[b53] Huang Z. A., Jiang D. A., Yang Y., Sun J. W. & Jin S. H. Effects of nitrogen deficiency on gas exchange, chlorophyll fluorescence, and antioxidant enzymes in leaves of rice plants. Photosynthetica 42, 357–364 (2004).

[b54] Elstner E. F. & Heupel A. Inhibition of nitrite formation from hydroxylammoniumchloride: a simple assay for superoxide dismutase. Anal. Biochem. 70, 616–620 (1976). 817618 10.1016/0003-2697(76)90488-7

[b55] Chance B. & Maehly A. Assay of catalases and peroxidases. Methods enzymol. 2, 764–775 (1955). 10.1002/9780470110171.ch14 13193536

[b56] Meng F. J., Peng M., Pang H. y. & Huang F. L. Comparison of photosynthesis and leaf ultrastructure on two black locust (*Robinia pseudoacacia* L.). Biochem.l Syst. Ecol. 55, 170–175 (2014).

[b57] Craven D., Gulamhussein S. & Berlyn G. Physiological and anatomical responses of *Acacia koa* (Gray) seedlings to varying light and drought conditions. Environ. Exp. Bot. 69, 205–213 (2010).

[b58] Makino A., Mae T. & Chira K. Colorimetric measurement of protein stained with coomassie brilliant blue ron sodium dodecyl sulfate–polyacrylamide gel electrophoresis by eluting with formamide. Agric. Biol. Chem. 50, 1911–1912 (1986).

[b59] Chen T. W. Kahlen K. & Stützel H. Disentangling the contributions of osmotic and ionic effects of salinity on stomatal, mesophyll, biochemical and light limitations to photosynthesis. Plant cell environ. doi: 10.1111/pce.12504 (2015). 25544985

