# Supplementary material for: Differences on photosynthetic limitations between leaf margins and leaf centers under potassium deficiency for Brassica napus L
Source: Sci Rep. 2016 Feb 23;6:21725. doi: 10.1038/srep21725 (PMC4763197; doi:10.1038/srep21725)
Supplement: Supplementary Information [file srep21725-s1.doc]

**Supplementary Information**

**Differences on photosynthetic limitations between leaf margins and leaf centers under potassium deficiency for *Brassica napus* L.**

Zhifeng Lu, Tao Ren, Yonghui Pan, Xiaokun Li, Rihuan Cong, Jianwei Lu*

**Supplementary Fig. S1** Leaf dry mass per unit area (*M*_A_) as affected by K deficiency.

**Supplementary Fig. S2** Transmission electron micrographs of leaf mesophyll cells of leaf margin and center in the fifth fully expanded leaves.

**Supplementary Fig. S3** The relationship between chloroplast number per cell and leaf K concentration. Values are mean±SE of at least thirty replicate cells.

**Supplementary Table S1** Sensitivity analysis of the influence of uncertainties in chloroplastic hypothetical CO_2_ compensation point (Γ*) and mitochondrial respiration rate in the light (*R*_d_) on the estimation of mesophyll conductance (*g*_m_).

**Supplementary Table S2** Sensitivity analysis of variable mesophyll conductance (*g*_m_) resulting from the biases of chloroplastic CO_2_ compensation point (Γ*) and mitochondrial respiration rate in the light (*R*_d_) on the estimation of photosynthetic limitations.

**Supplementary Table S3** Sensitivity analysis of variable mesophyll conductance (*g*_m_) response to *p*_1_ and *p*_2_ sets.

**Supplementary Table S4** Sensitivity analysis of variable mesophyll conductance (*g*_m_) resulting from the different *p*_1_ and *p*_2_ values on the estimation of photosynthetic limitations.

**Supplementary Table S5** Sensitivity of the estimation of mesophyll conductance (*g*_m_) for variation in electron transport rate (*J*) values.

**Supplementary Table S6** Sensitivity analysis of variable mesophyll conductance (*g*_m_) due to electron transport rate (*J*) biases on the estimation of photosynthetic limitations.


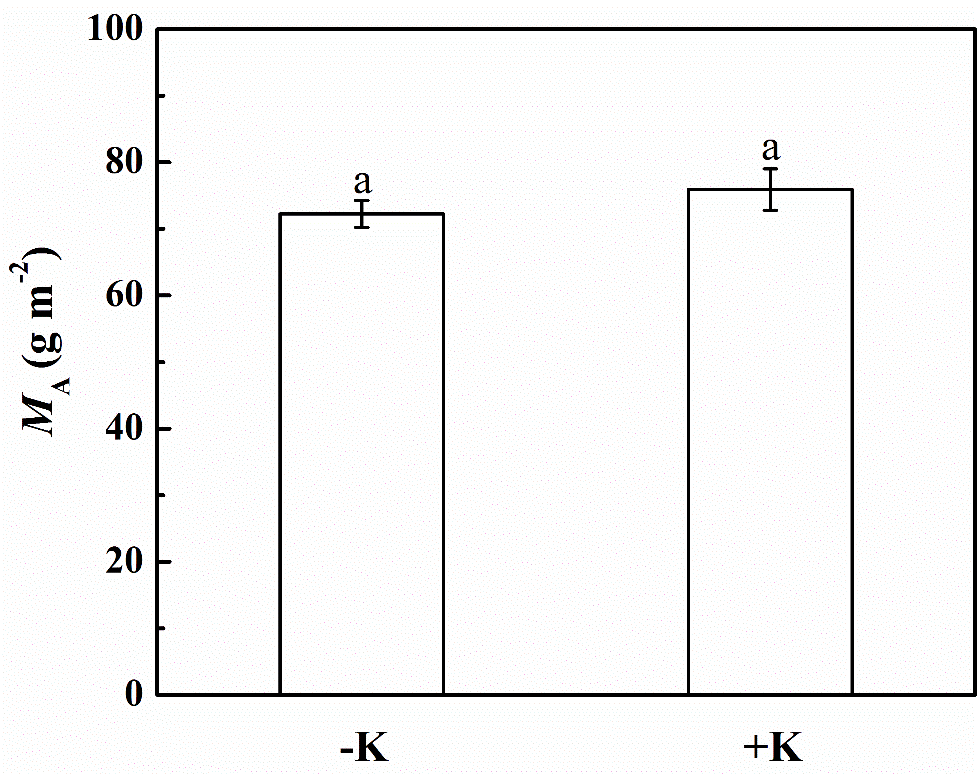


**Supplementary Fig. S1** Leaf dry mass per unit area (*M*_A_) as affected by K deficiency. Values are mean±SE of six replications. The same letters indicate no significant difference between treatments (*P*≤0.05).

**
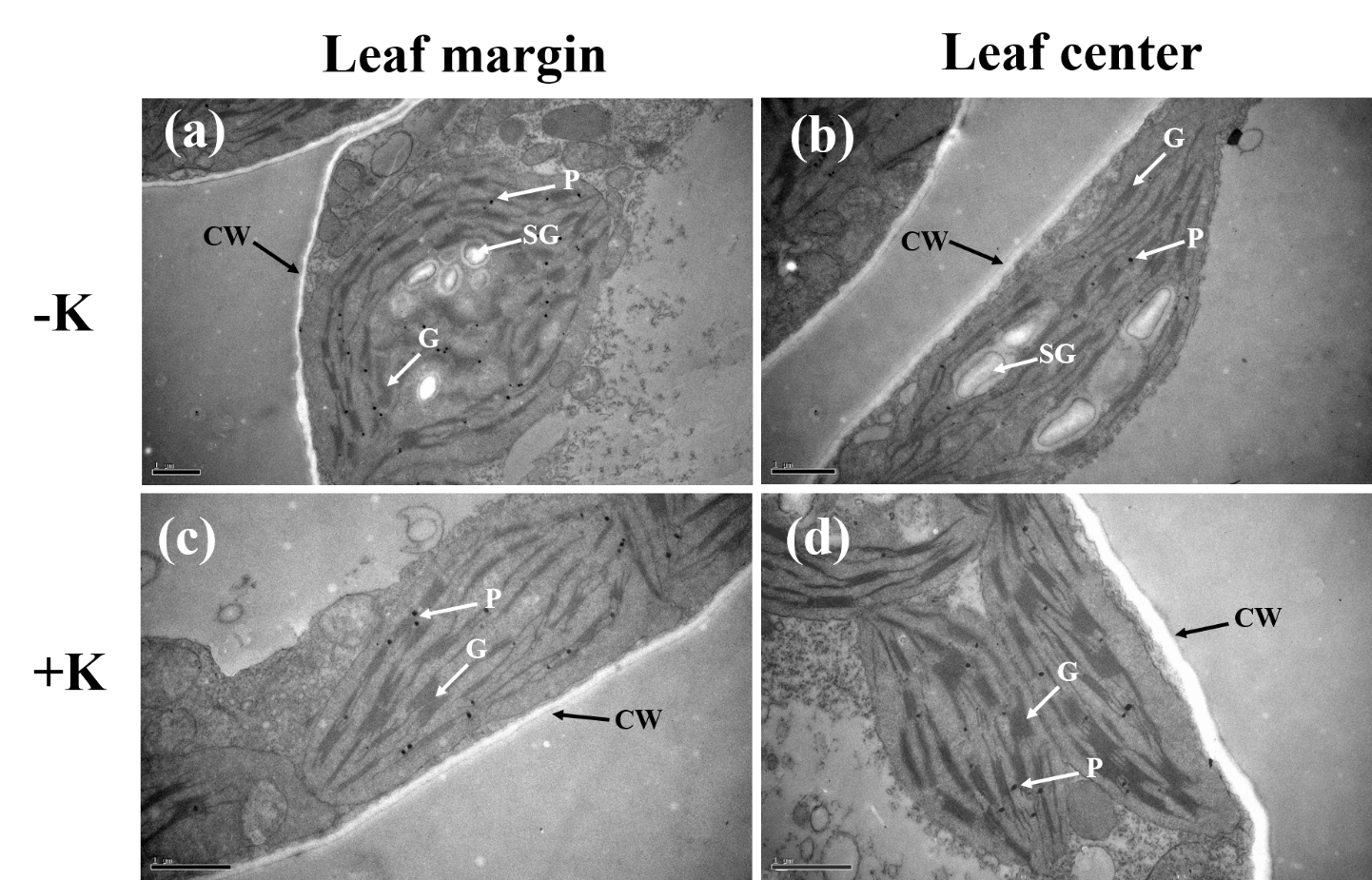
**

**Supplementary Fig. S2** Representative transmission electron micrographs of leaf mesophyll cells of leaf margin and center in the fifth fully expanded leaves. (**a**) Leaf margin under the –K treatment, (**b**) leaf center under the –K treatment, (c) leaf margin under the +K treatment, and (**d**) leaf center under the +K treatment. CW: cell wall, SG: starch granule, G: grana, P: plastoglobules. Bar=1μm. The detailed assay method is described later in the


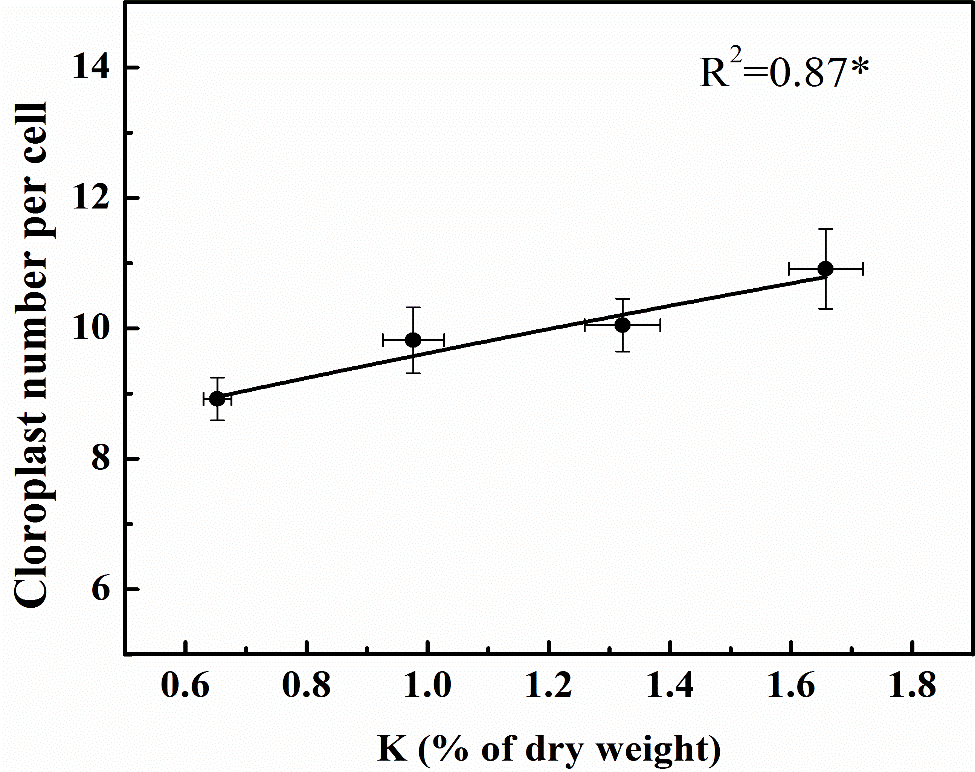


**Supplementary Fig. S3** The relationship between chloroplast number per cell and leaf K concentration. Values are mean±SE of at least thirty replicate cells. Regression coefficient and significance are shown when *P*≤0.05 (*, *P*≤0.05; **, *P*≤0.01).

**Supplementary Table S1** Sensitivity of the estimation of mesophyll conductance (*g*_m_) for variation in chloroplastic hypothetical CO_2_ compensation point (Γ*) and mitochondrial respiration rate in the light (*R*_d_) on the estimation of mesophyll conductance (*g*_m_). (A) *g*_m_ values were calculated by Harley *et al.* method using actual Γ* values calculated in this study and 10%, 5% elevated (or reduced) values^1^. (B) Mesophyll conductance calculated by Harley method using actual *R*_d_ calculated in this study and 50%, 25% elevated (or reduced) values.

| Treatment | Position | A | | | | |  | B | | | | |
| --- | --- | --- | --- | --- | --- | --- | --- | --- | --- | --- | --- | --- |
|  |  | (Γ*)  (mol CO_2_ m^-2^ s^-1^) | (1.10 Γ*)  (mol CO_2_ m^-2^ s^-1^) | (1.05 Γ*)  (mol CO_2_ m^-2^ s^-1^) | (0.95 Γ*)  (mol CO_2_ m^-2^ s^-1^) | (0.90 Γ*)  (mol CO_2_ m^-2^ s^-1^) |  | (*R*_d_)  (mol CO_2_ m^-2^ s^-1^) | (1.50 *R*_d_)  (mol CO_2_ m^-2^ s^-1^) | (1.25 *R*_d_)  (mol CO_2_ m^-2^ s^-1^) | (0.75 *R*_d_)  (mol CO_2_ m^-2^ s^-1^) | (0.50 *R*_d_)  (mol CO_2_ m^-2^ s^-1^) |
| -K | margin | 0.084±0.002ab^1^ | 0.099±0.008a | 0.092±0.009ab | 0.080±0.004b | 0.076±0.005b |  | 0.084±0.002ab | 0.091±0.003a | 0.088±0.002ab | 0.082±0.001b | 0.080±0.005b |
|  | center | 0.151±0.008bc | 0.183±0.009a | 0.168±0.009ab | 0.144±0.009bc | 0.134±.0011c |  | 0.151±0.007ab | 0.164±0.007a | 0.159±0.006ab | 0.150±0.002ab | 0.146±0.002b |
| +K | margin | 0.163±0.003bc | 0.207±0.009a | 0.183±0.012b | 0.149±.0.006cd | 0.137±0.004d |  | 0.163±0.003ab | 0.177±0.0013a | 0.174±0.005ab | 0.160±0.003ab | 0.153±0.004b |
|  | center | 0.174±0.018bc | 0.224±0.017a | 0.196±0.006ab | 0.157±0.018bc | 0.143±0.009c |  | 0.174±0.006ab | 0.187±0.007a | 0.180±0.007ab | 0.168±0.009ab | 0.163±0.002b |

Values are means±SE of three replicates per treatment.

^1^ Different letters donate significant differences at *P*<0.05 between *g*_m_ values obtained in this study (Γ* and *R*_d_) and each values estimated by different values of Γ^*^ and *R*_d_.

**Supplementary Table S2** Sensitivity analysis of variable mesophyll conductance (*g*_m_) resulting from the biases of chloroplastic CO_2_ compensation point (Γ*) and mitochondrial respiration rate in the light (*R*_d_) on the estimation of photosynthetic limitations (S_L_, stomatal limitation; MC_L_, mesophyll conductance limitation; B_L_, biochemical limitation; T_L_ (T_L_= S_L_+MC_L_+B_L_), total limitation). (A) Quantitative photosynthetic limitations calculated by Grassi and Magnani method using *g*_m_ values listed in Table S1A as affected by Γ^*^ biases^2^. (B) Quantitative photosynthetic limitations calculated by Grassi and Magnani method using *g*_m_ values listed in Table S1B as affected by *R*_d_ biases.

| Position | Limitation | A | | | | |  | B | | | | |
| --- | --- | --- | --- | --- | --- | --- | --- | --- | --- | --- | --- | --- |
|  |  | Γ*  (%) | 1.10 Γ*  (%) | 1.05 Γ*  (%) | 0.95 Γ*  (%) | 0.90 Γ*  (%) |  | *R*_d_  (%) | 1.50 *R*_d_  (%) | 1.25 *R*_d_  (%) | 0.75 *R*_d_  (%) | 0.50 *R*_d_  (%) |
| leaf margin | S_L_ | 11.20±0.66a^1^ | 11.65±0.83a | 11.47±0.80a | 11.13±0.74a | 10.97±0.72a |  | 11.20±0.66a | 11.55±0.68a | 11.47±0.83a | 11.32±0.79a | 11.24±0.77a |
|  | MC_L_ | 15.51±0.32a | 15.29±0.44a | 15.07±0.49a | 14.80±0.63a | 16.16±0.68a |  | 15.51±0.32a | 14.30±0.89a | 14.52±0.30a | 14.90±0.59a | 15.08±0.52a |
|  | B_L_ | 20.23±1.53a | 20.95±1.59a | 20.68±1.64a | 20.18±1.71a | 19.95±1.03a |  | 20.23±1.53a | 20.94±1.58a | 20.78±1.63a | 20.47±1.17a | 20.33±1.54a |
|  | T_L_ | 46.94±1.32a | 47.89±3.64a | 47.23±3.64a | 46.12±2.59a | 47.08±3.49a |  | 46.94±1.32a | 46.79±2.98a | 46.76±1.66a | 46.69±3.03a | 46.65±2.79a |
| leaf center | S_L_ | 0.95±0.26a | 0.97±0.46a | 0.59±0.24a | 0.56±0.19a | 0.55±0.16a |  | 0.95±0.26a | 0.91±0.25a | 0.91±0.31a | 0.89±0.27a | 0.89±0.32a |
|  | MC_L_ | 3.43±1.63a | 3.97±0.96a | 3.85±1.77a | 3.41±1.54a | 4.36±0.79a |  | 3.43±1.63a | 3.39±0.89a | 3.32±1.39a | 3.16±0.86a | 3.08±1.13a |
|  | B_L_ | 0.37±0.45a | 0.33±0.18a | 0.32±0.10a | 0.31±0.14a | 0.30±0.21a |  | 0.37±0.45a | 0.33±0.14a | 0.32±0.14a | 0.32±0.24a | 0.31±0.19a |
|  | T_L_ | 4.75±1.42a | 5.27±1.22a | 4.76±1.22a | 4.28±1.20a | 5.21±1.18a |  | 4.75±1.42a | 4.63±1.66a | 4.55±1.60a | 4.37±1.63a | 4.29±1.47a |

Values are means±SE of three replicates per treatment.

^1^ Different letters donate significant differences at *P*≤0.05 between limitation values obtained in this study (using *g*_m_ values based on Γ* and *R*_d_) and each values estimated by different values of *g*_m_ based on Γ^*^ and *R*_d_ biases.

**Supplementary Table S3 Sensitivity analysis of mesophyll conductance (*g*_m_) response to *p*_1_ and *p*_2_ sets. RuBP regeneration is limited by either insufficient NADPH (*p*_1_=4 and *p*_2_=8) or insufficient ATP (*p*_1_=4.5 and *p*_2_=10.5 or *p*_1_=4 and *p*_2_=9.33).**

| Treatment | Position | *g*_m_ (*p*_1_=4, *p*_2_=8) (mol CO_2_ m^-2^ s^-1^) | *g*_m_ (*p*_1_=4, *p*_2_=9.33) (mol CO_2_ m^-2^ s^-1^) | *g*_m_ (*p*_1_=4.5, *p*_2_=10.5) (mol CO_2_ m^-2^ s^-1^) |
| --- | --- | --- | --- | --- |
| -K | margin | 0.084±0.002b^1^ | 0.090±0.004b | 0.107±0.007a |
|  | center | 0.151±0.008b | 0.194±0.025b | 0.312±0.0019a |
| +K | margin | 0.163±0.003c | 0.217±0.012b | 0.331±0.011a |
|  | center | 0.174±0.018c | 0.222±0.014b | 0.333±0.032a |

Values are means±SE of four replicates per treatment.

^1^ Different letters donate significant differences at *P*≤0.05 between *g*_m_ values obtained in this study (*p*_1_=4, *p*_2_=8) and each values estimated by different *p*_1_ and *p*_2_ inputs.

**Supplementary Table S4 Sensitivity analysis of variable mesophyll conductance (*g*_m_) resulting from the different *p*_1_ and *p*_2_ values on the estimation of photosynthetic limitations (S_L_, stomatal limitation; MC_L_, mesophyll conductance limitation; B_L_, biochemical limitation; T_L_ (T_L_= S_L_+MC_L_+B_L_), total limitation). Quantitative photosynthetic limitations calculated by Grassi and Magnani method using *g*_m_ values listed in Table S3 as affected by *p*_1_ and *p*_2_ sets.**

| Position | Limitation | *p*_1_=4, *p*_2_=8 (%) | *p*_1_=4, *p*_2_=9.33 (%) | *p*_1_=4.5, *p*_2_=10.5 (%) |
| --- | --- | --- | --- | --- |
| leaf margin | S_L_ | 11.20±0.66a^1^ | 11.43±0.75a | 11.76±0.69a |
|  | MC_L_ | 15.51±0.32a | 16.60±0.31a | 17.75±0.37a |
|  | B_L_ | 20.23±1.53a | 21.30±1.75a | 21.47±1.62a |
|  | T_L_ | 46.94±1.32a | 48.85±2.44a | 50.98±1.42a |
| leaf center | S_L_ | 0.95±0.26a | 1.20±0.33a | 1.37±0.15a |
|  | MC_L_ | 3.43±1.63a | 4.31±1.91a | 4.74±1.09a |
|  | B_L_ | 0.37±0.45a | 0.52±0.27a | 0.47±0.36a |
|  | T_L_ | 4.75±1.42a | 6.03±1.49a | 6.58±1.62a |

Values are means±SE of four replicates per treatment.

^1^ Different letters donate significant differences at *P*≤0.05 between limitation values obtained in this study (using *g*_m_ values based on *p*_1_=4, *p*_2_=8) and each values estimated by different values of *g*_m_ based on different *p*_1_ and *p*_2_ inputs.

**Supplementary Table S5 Sensitivity of the estimation of mesophyll conductance (*g*_m_) for variation in electron transport rate (*J*) values. *g*_m_ values were calculated by using actual *J* obtained in this article and 5%, 10% elevated (or reduced) values.**

| Treatment | Position | (*J*)  (mol CO_2_ m^-2^ s^-1^) | (1.10*J*)  (mol CO_2_ m^-2^ s^-1^) | (1.05*J*)  (mol CO_2_ m^-2^ s^-1^) | (0.95*J*)  (mol CO_2_ m^-2^ s^-1^) | (0.90*J*)  (mol CO_2_ m^-2^ s^-1^) |
| --- | --- | --- | --- | --- | --- | --- |
| -K | margin | 0.084±0.002ab^1^ | 0.075±0.007b | 0.079±0.008b | 0.089±0.011ab | 0.097±0.013a |
|  | center | 0.151±0.008bc | 0.126±0.012c | 0.136±0.014c | 0.174±0.024b | 0.204±0.012a |
| +K | margin | 0.163±0.003bc | 0.123±0.009d | 0.138±0.012cd | 0.185±0.016ab | 0.211±0.028a |
|  | center | 0.174±0.018abc | 0.134±0.029c | 0.149±0.038bc | 0.188±0.011ab | 0.226±0.025a |

Values are means±SE of four replicates per treatment.

^1^ Different letters donate significant differences at *P*<0.05 between *g*_m_ values obtained in this study (*J*) and each values estimated by different values of *J*.

**Supplementary Table S6 Sensitivity analysis of variable mesophyll conductance (*g*_m_) resulting from *J* biases on the estimation of photosynthetic limitations (S_L_, stomatal limitation; MC_L_, mesophyll conductance limitation; B_L_, biochemical limitation; T_L_ (T_L_= S_L_+MC_L_+B_L_), total limitation). Quantitative photosynthetic limitations calculated by Grassi and Magnani method using *g*_m_ values listed in Table S5 as affected by changed *J*.**

| Position | Limitation | (*J*)  (%) | (1.10*J*)  (%) | (1.05*J*)  (%) | (0.95*J*)  (%) | (0.90*J*)  (%) |
| --- | --- | --- | --- | --- | --- | --- |
| leaf margin | S_L_ | 11.20±0.66a^1^ | 10.97±0.67a | 11.07±0.68a | 11.35±0.71a | 11.53±0.75a |
|  | MC_L_ | 15.51±0.32a | 16.22±0.65a | 15.87±0.62a | 14.92±0.70a | 14.32±0.89a |
|  | B_L_ | 20.23±1.53a | 19.81±1.31a | 20.01±1.84a | 20.53±1.92a | 20.86±1.87a |
|  | T_L_ | 46.94±1.32a | 47.00±1.76a | 46.95±2.31a | 46.80±2.16a | 46.71±2.24a |
| leaf center | S_L_ | 0.95±0.26a | 0.89±0.21a | 0.92±0.36a | 0.99±0.41a | 1.03±0.29a |
|  | MC_L_ | 3.43±1.63a | 3.70±1.27a | 4.18±1.13a | 3.96±1.01a | 3.43±1.51a |
|  | B_L_ | 0.37±0.45a | 0.35±0.22a | 0.36±0.19a | 0.38±0.48a | 0.40±0.30a |
|  | T_L_ | 4.75±1.42a | 4.94±2.01a | 5.46±1.52a | 5.32±1.14a | 4.87±1.09a |

Values are means±SE of four replicates per treatment.

^1^ Different letters donate significant differences at *P*≤0.05 between limitation values obtained in this study (using *g*_m_ values based on *J*) and each values estimated by different values of *g*_m_ based on *J* biases.

**References:**

1. Harley, P.C., Loreto, F., Di Marco, G. & Sharkey, T.D. Theoretical considerations when estimating the mesophyll conductance to CO_2_ flux by analysis of the response of photosynthesis to CO_2_. *Plant Physiol.* **98,** 1429-1436 (1992).

2. Grassi, G. & Magnani, F. Stomatal, mesophyll conductance and biochemical limitations to photosynthesis as affected by drought and leaf ontogeny in ash and oak trees. *Plant Cell Environ.* **28,** 834-849 (2005) .
